# Unveiling of the epidemiological patterns for caprine/ovine enterovirus infection in China

**DOI:** 10.3389/fvets.2022.1025916

**Published:** 2022-11-28

**Authors:** Junying Hu, Xiaoran Chang, Rudu Wang, Qun Zhang, Fan Zhang, Zhiyuan Zhang, Fuhui Zhang, Mingzhu Qian, Xinping Wang

**Affiliations:** ^1^State Key Laboratory for Zoonotic Diseases, Key Laboratory of Zoonosis Research of Ministry of Education, College of Veterinary Medicine, Jilin University, Changchun, China; ^2^College of Animal Husbandry Engineering, Henan Agricultural Vocational College, Zhengzhou, China

**Keywords:** caprine/ovine enterovirus infection, CEV/OEV, goat/sheep, epidemic pattern, epidemiology, contribution factors

## Abstract

Caprine/ovine enterovirus (CEV/OEV) infection is an emerging disease and remains largely unknown for its infection distribution, epidemic pattern, and the underlying contribution factors. Here, we report the investigation on CEV/OEV infection pattern and the underlying contribution factors by employing a sandwich ELISA kit for detection of CEV/OEV antigen. Epidemiological investigation revealed a wide range of infection rates of CEV/OEV from 19.80%−39.00% on goat/sheep farms in the major goat/sheep-raising provinces as such Henan, Shandong, Ningxia, Jilin, Inner Mongolia autonomous region, and Xinjiang autonomous region in China. Epidemic patterns and infection rates for CEV/OEV were affected by the breeds, raising mode, regions, and seasons. CEV/OEV infection rates were varied in different regions in China and significantly higher in the diarrheal herds (40.30%) than these in non-diarrheal herds (13.83%). Moreover, infection rate was higher in sheep (24.59%) than that in goats (9.84%), even dramatic difference among different breeds of goat or sheep. Out of different breeds of goat, Boer (20.13%) had the highest infection rate, followed by local breed (5.62%) and Saanen (2.61%). Among these breeds of sheep, higher infection rates were detected in local breed sheep (42.86%) and small-tailed Han sheep (35.91%) than these of Hu sheep (13.41%) and Dorper sheep (16.34%). Furthermore, raising modes were showed to contribute to the infection rate, where higher rates were detected among goats/sheep in captivity (27.10%) than these in free-range (12.27%) and semi-free range (19.24%). Additionally, CEV/OEV infection rate had obvious seasonality, while they increased from year 2015 to 2019. In summary, we investigated the CEV/OEV infection among the goat/sheep herds from different regions in China, revealed the epidemic pattern and the contribution factors to the infection, which provided the epidemiological data for future prevention and control of this emerging infection.

## Introduction

Caprine/ovine enterovirus (CEV/OEV) infection is the recently reported disease characterized by digestive and respiratory signs in goats or sheep (1–4). As the causative agent, CEV/OEV belongs to the genus of *Enterovirus* within the family of *Picornaviridae*. According to the latest virus classification by the International Committee on Taxonomy of Viruses, the genus of *Enterovirus* consists of 12 species of enterovirus and 3 species of rhinovirus (5, 6). Out of 12 species of enterovirus, enterovirus E (EV-E) and enterovirus F (EV-F) mainly contribute to cattle infection (7–15), while enterovirus G (EV-G) is associated with pig infection (16–19). Recently, CEV/OEV infections were increasingly reported, and several strains of CEV/OEV isolated from goats or sheep were also classified as EV-G. In, Boros, et al. (1) reported the complete genome sequence of an ovine enterovirus strain TB4-OEV. Later on, Wang, et al. (3) reported the isolation of the first caprine enterovirus strain CEV-JL14 from goats with severe diarrhea, and unveiled its complete genome sequence. The discovery of EV-F infection in goats and the existence of co-infection of EV-F with EV-G in the same goat herd broadened the understanding of host range for EV-F infection (20). Currently, CEV/OEV strains were grouped to three types (EV-G5, EV-G7 and EV-G20) in EV-G, where CEV-JL14 isolate was designated as a reference strain for EV-G20 (5, 19). Chang et al. reported the molecular difference of the caprine/ovine enterovirus strains circulating in China and revealed 3 new EV-G types for CEV/OEV viruses based on the VP1 sequence variations, which were proposed to be named EV-G21, EV-G22, and EV-G23 (21).

Little information was available on CEV/OEV infection and its epidemiological aspects until the establishment of a sandwich ELISA method for CEV antigen detection as recently reported (22). More recently, Dong et al. reported the establishment of a RT-PCR method for detecting caprine/ovine enterovirus, which is also suitable for rapid diagnosis of enterovirus infection in clinical samples (23). Although some progress was made on the CEV/OEV infection, the geographic distribution and epidemic pattern for CEV/OEV infection remain largely unknown. In this study, we conducted an epidemiological investigation on caprine/ovine enterovirus infection in China by detecting the CEV/OEV antigens in the specimens collected from six major goat/sheep-raising provinces in China during 2014-2021, explored the factors contributing to CEV/OEV infection, and revealed a wide enterovirus infection in different goat/sheep herds, thus enriching the current understanding on the epidemiology aspects for CEV/OEV infection.

## Materials and methods

### Sample collection and processing

**Figure 1:**
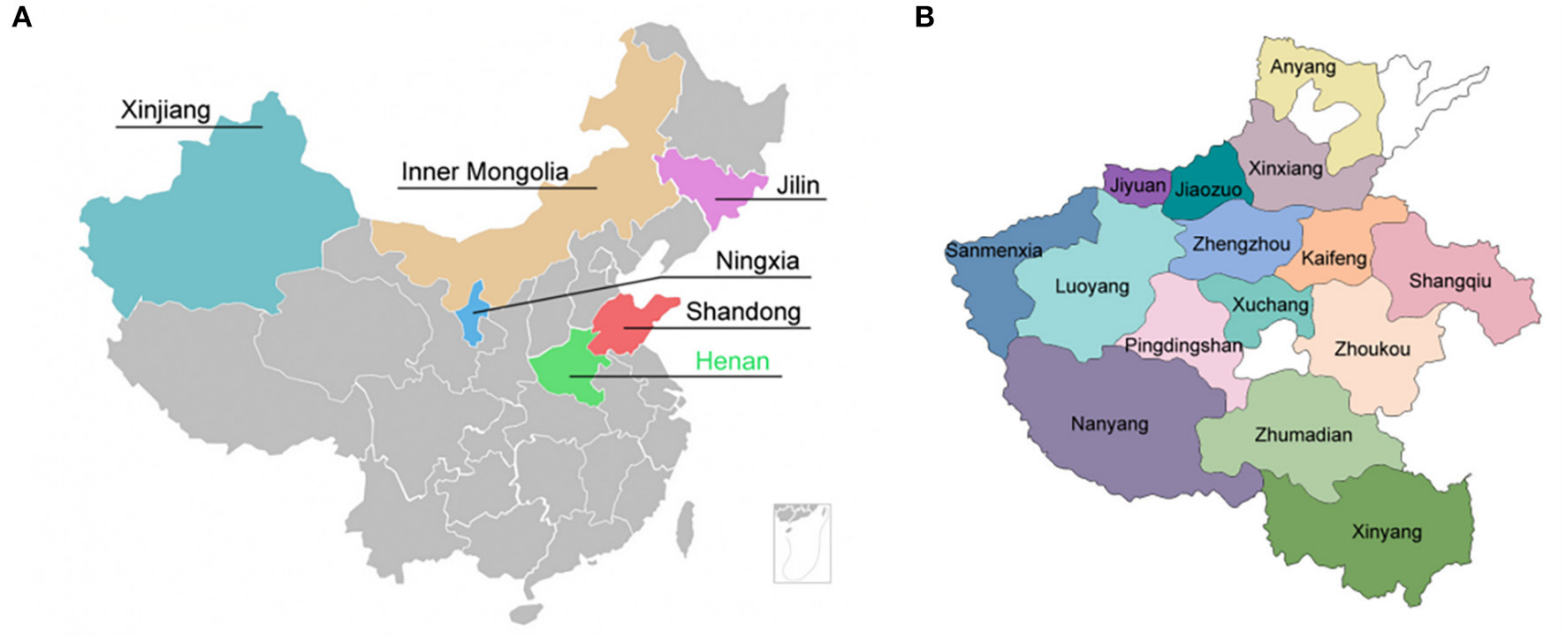
Geographic localization for specimens collection. **(A)** Six provinces in China where specimens were collected. **(B)** 15 regions in Henan Province where specimens were collected.

A total of 3,780 fecal samples was collected from goat/sheep herds in six provinces in China including Henan, Shandong, Ningxia, Jilin, Inner Mongolia, and Xinjiang (Figure 1A). Out of 3,780 specimens, 1,907 fecal specimens were collected from 15 regions in Henan Province from 2015 to 2019 (Figure 1B). The goat/sheep herds were randomly selected for specimen collection. Each specimen was recorded following a standard epidemiological investigation procedure including sample region, status of herds, status of feces, collection season, raising mode, immunization status, and disease history.

Fecal swab samples were processed as previously reported (3, 14). In short, the samples were diluted with 10 mM phosphate buffered saline (PBS) (pH 7.4) at a dilution of 1:10 (W/V) and homogenized before centrifugation at 12,000 × g for 10 min at 4°C. The supernatants were collected and stored at−20°C for later usage.

### Detection of CEV/OEV antigen using sandwich ELISA kit

The processed samples were assayed by the double-antibody sandwich ELISA detection kit for CEV/OEV following the procedures as previously reported (22). Specimens were added to the pre-blocked ELISA reaction plates coated with anti-CEV-VP1 mAb as capture antibody, and incubated at 37°C for 60 min. After washing with 10 mM phosphate buffered saline (pH 7.4), 0.05% Tween-20 (PBS-T), HRP-conjugated rabbit pAb against CEV-VP1 (1:1 000 dilution) was added and incubated at 37°C for 45 min. After washing, the substrate solution (OPD) was added and kept at room temperature for 15 min before terminating the reaction using 2 M H_2_SO_4_. OD_490_ was measured and recorded.

### Amplification of the virus gene by RT-PCR

Total RNA was extracted from the samples using TRIzol™ reagent (Invitrogen, Carlsbad, CA) following manufacturer's instructions. Briefly, samples were lysed with TRIzol™ reagent, mixed well with 0.2 volume of chloroform, and centrifuged at 12,000 × g for 15 min at 4°C. The aqueous phase was mixed with an equal volume of isopropanol and centrifuged at 12,000 × g for 10 min at 4°C. The RNA precipitate was washed with 75% ethanol, dried and dissolved in RNase free H_2_O.

Reverse transcriptase reaction was carried out using Reverse Transcriptase M-MLV (RNase H-) (TaKaRa, Dalian, China). Briefly, cDNA was synthesized in a volume of 10 μL containing 1 μg of total RNA, 25 μM random primers, 2.5 mM dNTP mixture, 200 units of Reverse Transcriptase M-MLV (RNase H-), 5 × Reverse Transcriptase M-MLV Buffer, 40 units of RNase inhibitor, and RNase free H_2_O. The cDNA synthesis was performed at 42°C for 60 min.

PCR amplification was performed using Premix Taq™ DNA polymerase (TaKaRa). The reaction was performed in an indicated volume of solution containing 1.25 units of Premix Taq™ DNA polymerase, 1 μM of each primer, 500 ng of the above synthesized cDNA, and RNase free H_2_O. PCR amplification was performed with the optimized conditions. The primer sequences for virus detection were listed as following. CEV-UP 5'- CTTTGCACGCCTGTTTTCC−3'; CEV-DN 5'- CACACGCTCGGAGGTTGGGATTAG -3'.

### Virus isolation and observation

Vero cells were grown in Dulbecco's modified Eagle medium (DMEM) (Invitrogen) containing 10% fetal bovine serum (FBS) (HyClone, Logan, UT), 2 mM L-glutamine, and 2 μg/mL gentamycin, and maintained in DMEM containing 2% FBS after inoculating the samples. For virus isolation, samples were processed with PBS and then filtered with 0.45 μm filters before infecting cells. After infection, cell cultures were observed for cytopathic effect (CPE) in every 6 h. The non-infected cells served as negative control.

Electron microscopy examination was performed by incubation of the processed samples with 1% phosphotungstic acid. The virus particle was pictured using EM (JEOL, Tokyo, Japan).

### Identification of virus by immunoperoxidase monolayer assay

The virus-infected Vero cells were fixed with methanol/acetone (1:1) at−20°C for 30 min. After blocking with 5% non–fat milk for 1 h, the polyclonal antibody against CEV-VP1 protein (1:500 dilution) was incubated at 37°C for 1 h, followed by incubation of HRP-conjugated goat anti-rabbit IgG (Abcam, Cambridge, United Kingdom) (1:1,000 dilution) at 37°C for 45 min. The cells were observed under the Canon digital camera (Canon, Tokyo, Japan) after they were stained with 3-amino-9-ethylcarbazole substrate (Amresco, Olympia, WA, Unite States).

### Data analysis

Infection rates and infection pattern for CEV/OEV were analyzed based on the results from samples collected from different regions, clinical signs, years, goat or sheep breeds, feeding modes, and seasons.

### Statistical analysis

All data were analyzed statistically using the software SPSS. Infection rates were compared between different groups using the Chi-square test or Fisher's exact test to determine whether the significant difference was existed in two sampling populations. The value ^*^p < 0.05 and ^**^p < 0.01 represent significant and highly significant differences, respectively.

## Results

### Caprine/ovine enterovirus infection in different regions in China

To determine the CEV/OEV infection in goat/sheep herds in China, samples collected from six provinces were used for detection of CEV/OEV antigens using the CEV antigen detection kit previously reported (22). As shown in Table 1, CEV/OEV infections were detected in all six provinces with a varied infection rate from 19.80 to 39.00%. The infection rate in Xinjiang was up to 39.00%, followed by 32.04% in Inner Mongolia, 25.70% in Ningxia, 24.51% in Jilin, 21.13% in Henan, and 19.80% in Shandong province. These results demonstrated a wide enterovirus infection in goat/sheep herds in China.

**Table 1:** Infection rates of CEV/OEV in different regions in China.

**Region**	**Total number**	**Positive number**	**Infection rate (%)**
Henan	1,907	403	21.13
Shandong	490	97	19.80
Ningxia	214	55	25.70
Jilin	657	161	24.51
Inner Mongolia	412	132	32.04
Xinjiang	100	39	39.00

### CEV/OEV infection revealed in different regions of Henan Province

Since the goat/sheep breeding industry in Henan Province ranks top in China, it is natural to investigate systematically the CEV/OEV infection in Henan Province to better understand the epidemiologic pattern on CEV/OEV infection. Total of 1,907 fecal specimens from 15 prefectures in Henan Province from 2015 to 2019 were collected and used to detect enterovirus infection. As shown in Table 2, out of 1,907 specimens, 403 specimens were detected as CEV/OEV-positive, with an average infection rate of 21.13%. Further analysis showed CEV/OEV infections existed in all 15 prefectures in Henan province with much severe infections in regions as such Luoyang and Anyang. The CEV/OEV infection rate in Luoyang was up to 33.65%, followed by Anyang of 32.46%. The CEV/OEV infection rate in Pingdingshan was the lowest, only of 10.85%.

**Table 2:** CEV/OEV infection in different regions in Henan Province.

**Region**	**Total specimens**			**Diarrheal specimens**			**Non-diarrheal specimens**		
	**Total number**	**Positive number**	**Infection rate (%)**	**Total number**	**Positive number**	**Infection rate (%)**	**Total number**	**Positive number**	**Infection rate (%)**
Zhengzhou	482	75	15.56	152	42	27.63	330	33	10.00
Kaifeng	162	42	25.93	51	32	62.75	111	10	9.01
Luoyang	211	71	33.65	31	24	77.42	180	47	26.11
Anyang	114	37	32.46	29	22	75.86	85	15	17.65
Xuchang	71	18	25.35	21	8	38.10	50	10	20.00
Zhoukou	73	15	20.55	22	12	54.55	51	3	5.88
Nanyang	86	14	16.28	23	8	34.78	63	6	9.52
Zhumadian	74	18	24.32	19	9	47.37	55	9	16.36
Sanmenxia	81	15	18.52	18	7	38.89	63	8	12.70
Xinyang	74	21	28.38	21	9	42.86	53	12	22.64
Jiyuan	86	19	22.09	23	9	39.13	63	10	15.87
Xinxiang	112	16	14.29	26	8	30.77	86	8	9.30
Pingdingshan	129	14	10.85	38	8	21.05	91	6	6.59
Shangqiu	78	19	24.36	21	7	33.33	57	12	21.05
Jiaozuo	74	9	12.16	31	7	22.58	43	2	4.65
Total	1,907	403	21.13	526	212	40.30	1,381	191	13.83

### Higher CEV/OEV infection rates revealed in diarrheal specimens than in non-diarrheal specimens

**Figure 2:**
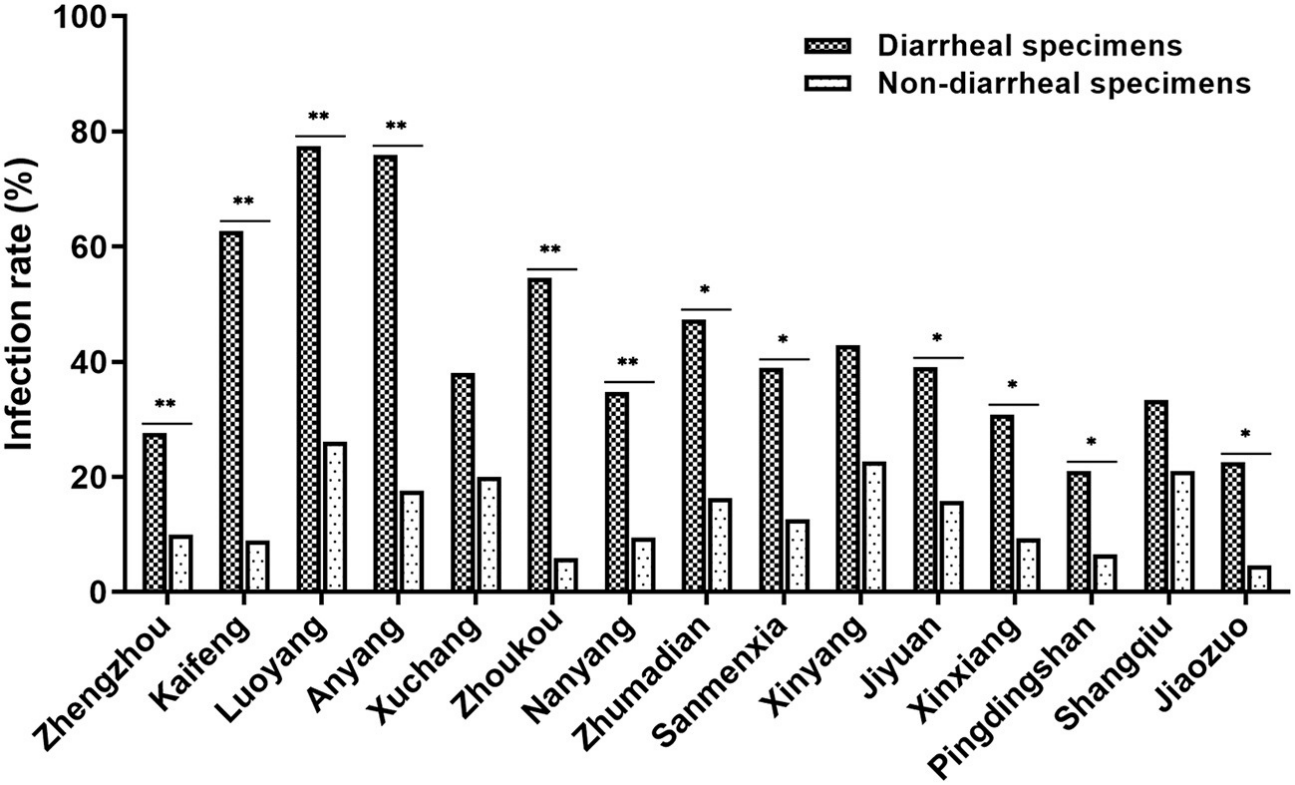
CEV/OEV infection rates in diarrheal specimens and non-diarrheal specimens from different regions in Henan Province. The CEV/OEV infection rates in diarrheal specimens and non-diarrheal specimens were detected and comparatively analyzed. Dark histogram (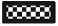) represents the CEV/OEV infection rates in diarrheal specimens, while the light bar chart (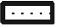) represents the CEV/OEV infection rates in non-diarrheal specimens. *Indicates the significant difference between the rates (*p* < 0.05), while **indicates highly significant difference (*p* < 0.01).

To determine whether CEV/OEV infection rate had any difference between diarrheal and non-diarrheal goats/sheep, 526 specimens from diarrheal goats/sheep and 1,381 specimens from non-diarrheal goats/sheep were used for enterovirus detection. As shown in Table 2, a total of 212 diarrheal samples and 191 non-diarrheal samples were detected as CEV/OEV positive, with an infection rate of 40.30% and 13.83%, separately. Out of the diarrheal samples, the CEV/OEV infection rate in Luoyang region reached 77.42%, followed by Anyang region of 75.86%. The infection rate in diarrheal specimens from Pingdingshan was only 21.05%, much lowered in comparison with these from other regions. Out of the non-diarrheal specimens, the infection rate of Luoyang was 26.11%, followed by Xinyang of 22.64%. The infection rate in Jiaozuo was 4.65%, the lowest in non-diarrheal specimens. Statistical analysis showed that the total CEV/OEV infection rate of diarrheal samples (40.30%) was significantly higher than that of non-diarrheal samples (13.83%). As illustrated in Figure 2, CEV/OEV infection rates in diarrheal samples from different regions in Henan Province were also mostly higher than those in non-diarrheal samples. These results revealed the CEV/OEV infection in both diarrheal herds and non-diarrheal herds, indicating CEV/OEV is likely one of the causative agents leading to diarrhea.

### Increase of the CEV/OEV infection rate in goat/sheep herds

**Figure 3:**
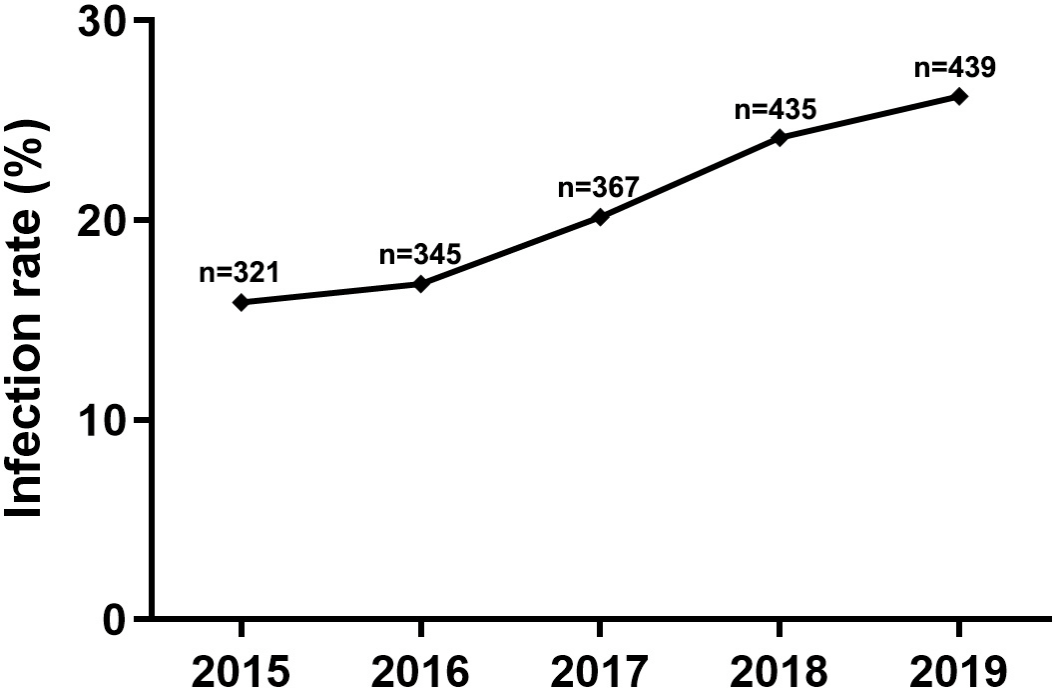
CEV/OEV infection rates of specimens in different years. The node represents the CEV/OEV infection rate for corresponding year from 2015 to 2019. “n” represents the number of specimens detected.

To determine the difference of CEV/OEV infection rate among different years, specimens collected during 2015–2019 were comparably analyzed. As illustrated in Figure 3, CEV/OEV infection rates in 2015, 2016, 2017, 2018, and 2019 were 15.89, 16.81, 20.16, 24.14, and 26.20%, respectively. CEV/OEV infection rate in 2019 was higher than these in 2015, 2016, 2017, and 2018, with an increase rate by 10.31, 9.39, 6.04, and 2.06%, respectively. These results suggest that CEV/OEV infection had tendency to increase in Henan province in recent years.

### Breed matter on CEV/OEV infection

**Figure 4:**
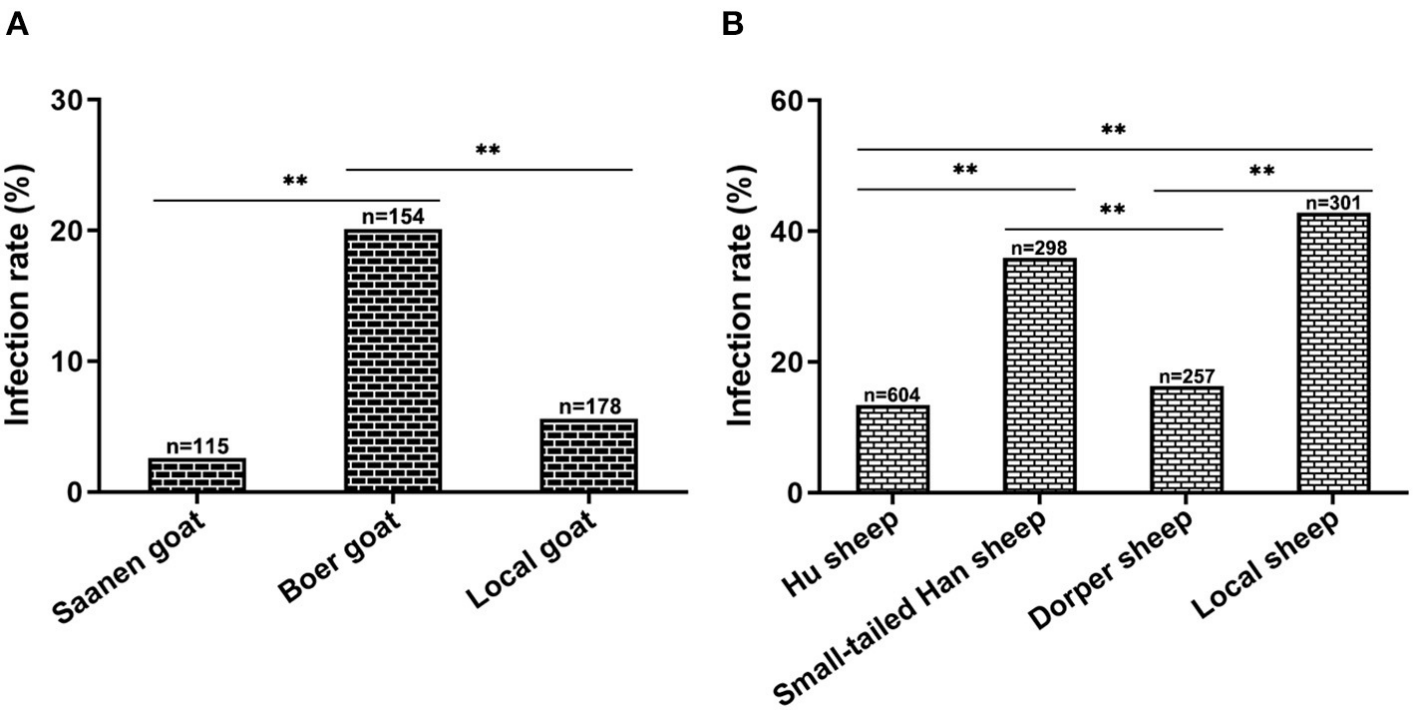
Influence of goat/sheep breed on CEV/OEV infection. **(A)** Effect of goat breed on CEV infection. CEV infection rates of different breeds were comparatively analyzed. The CEV infection rate of Boer goats was much higher than that of Saanen goats and local goats (*p* < 0.01), while there was no significant difference between Saanen goats and local goats (*P* > 0.05). **(B)** Influence of sheep breed on OEV infection. OEV infection rates of small-tailed Han sheep and local sheep were revealed significantly higher than those of Hu sheep and Dorper sheep (*p* < 0.01), while no significant differences were observed between small-tailed Han sheep and local sheep (*P* > 0.05), as well as Hu sheep and Dorper sheep (*P* > 0.05). “n” represents the number of specimens detected. **Indicates highly significant difference (*P* < 0.01).

To explore whether the breed of goats or sheep has any influence on the CEV/OEV infection, infection rates of different breeds of goats or sheep were analyzed. As shown in Figure 4, the infection rate in goat was 9.84%, while the infection rate in sheep was 24.59%, indicating that sheep was more susceptible than goat. In goats, infection rates were significantly different among breeds of goats, where Boer had the highest CEV-infection rate of 20.13%, followed by local goat breed of 5.62% and Saanen goat of 2.61% (Figure 4A). Similarly, among sheep breeds, the infection rate was the highest in local sheep, reaching 42.86%, followed by small-tailed Han sheep of 35.91%. The infection rates were relatively lower in Hu sheep and Dorper sheep at 13.41% and 16.34%, respectively (Figure 4B).

Taken together, the above results demonstrated the enterovirus infection in goat and sheep, revealed the influence of breed on CEV/OEV infection.

### Influence of CEV/OEV infection by raising modes and seasons

**Figure 5:**
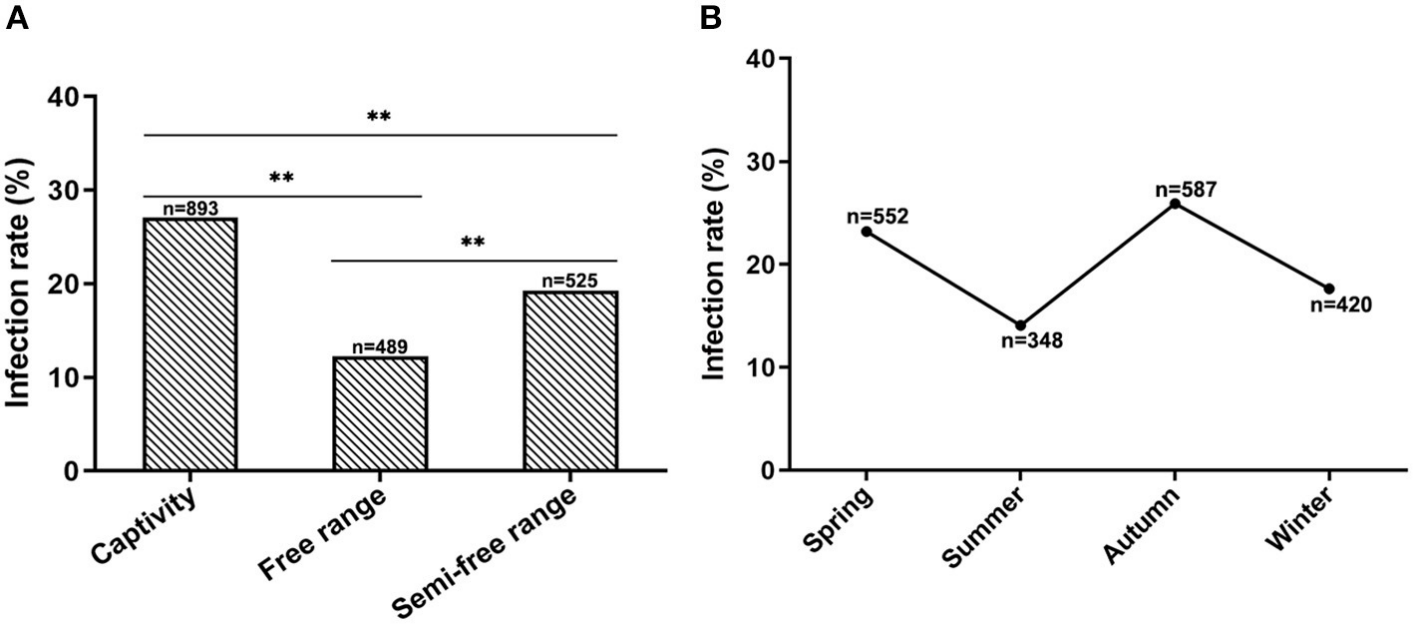
Effect of feeding modes and seasons on CEV/OEV infection. **(A)** CEV/OEV infection rates in different feeding modes. CEV/OEV infection rates of different feeding modes were detected and comparatively analyzed. The CEV/OEV infection rate of goats/sheep in captivity was significantly higher than that of goats/sheep in free range and semi-free range, while higher infection rate of goats/sheep was observed in semi-free range than in free range. **(B)** CEV/OEV infection rates in four seasons. CEV/OEV infection rates of different seasons were comparatively analyzed. The infection rates were higher in spring and autumn than those in summer and winter. “n” represents the number of specimens detected. **Indicates highly significant difference (*P* < 0.01).

To investigate if the CEV/OEV infection is affected by raising modes, specimens collected from goats and sheep raised in different feeding modes were examined and analyzed. As shown in Figure 5A, the infection rate of goats/sheep in captivity was 27.10%, which is significantly higher that these goats/sheep in free-range (12.27%) and semi-free range (19.24%), indicating that feeding modes did affect CEV/OEV infection rates.

To determine whether CEV/OEV infection has any seasonality, fecal samples collected in Spring, Summer, Autumn and Winter from the goat/sheep herds were used for enterovirus detection. As shown in Figure 5B, the CEV/OEV infection rates in samples collected in Spring, Summer, Autumn, and Winter were revealed as 23.19, 14.08, 25.89, and 17.62%, respectively. Statistical analysis showed the infection rates among different seasons have significant differences, suggesting that CEV/OEV infection has obvious seasonality, and the infection rate was higher in Spring and Autumn than that in Summer and Winter.

### Confirmation of CEV/OEV infection by RT-PCR and electron microscopy

**Figure 6:**
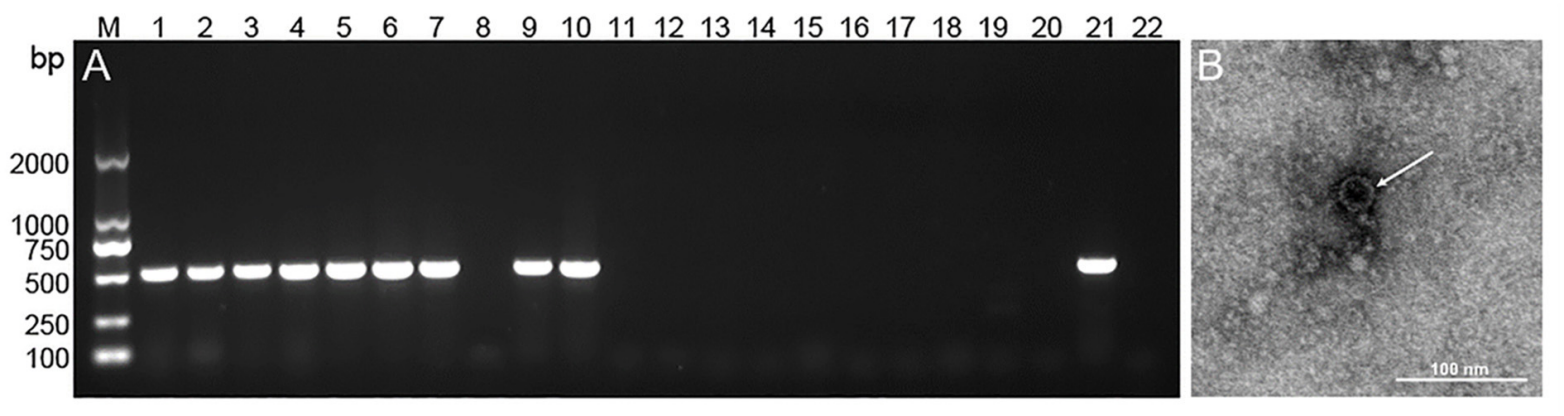
Representative figure showing the confirmation of CEV/OEV infection in specimens by RT-PCR and electron microscopy. **(A)** PCR amplification of the representative ELISA-detected positive (lane 1–10) and negative (line 11–20) specimens with a positive (lane 21) and negative control (line 22). **(B)** Observation of enterovirus particle in ELISA-detected positive specimens by electron microscopy.

To confirm the CEV/OEV infection revealed by double antibody sandwich ELISA kit, 10 positive and 10 negative specimens were selected for RT-PCR amplification. As shown in Figure 6, the fragments with the expected size were amplified from 9 out of 10 positive samples, while no gene fragments were amplified from all 10 negative samples. Sequencing confirmed the amplified fragment containing enterovirus sequences. The compliance rate of the two tests for CEV/OEV detection was 95.0%. Electron microscopy observation revealed the typical enterovirus particles in ELISA-positive samples, while no enterovirus particles were observed in ELISA-negative specimens. The compliance rate of ELISA with electron microscopy was 100%.

### Confirmation of CEV/OEV isolates by IPMA

**Figure 7:**
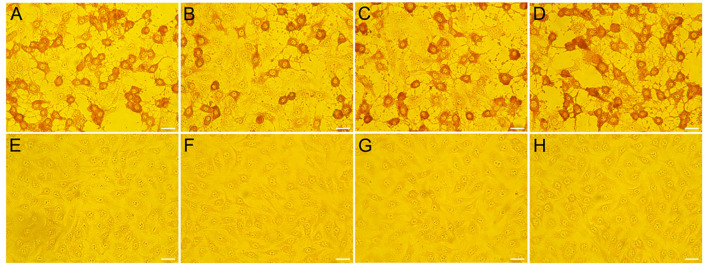
Confirmation of the representative CEV/OEV virus strain by IPMA. Vero cells infected with specimens were incubated with polyclonal antibody raised in rabbit against CEV-VP1 protein. Strong immunoreactions were observed in the cells inoculated with positive samples (HeN-T3-12, HeN-D1-37, and HeN-D2-57) **(A–C)**, while no reactions were observed in the cells inoculated with negative samples **(E–G)**. Vero cells infected by CEV-JL14 **(D)** and non-infected cells **(H)** were used as positive and negative controls, respectively. Bar = 20 μm.

To further confirm the ELISA results, Vero cells were used to isolate potential viruses from 30 ELISA-positive fecal samples collected from Henan province. After virus isolation, three strains were isolated and named as HeN-T3-12, HeN-D1-37, and HeN-D2-57, respectively. Virus-infected cells were incubated with antibody generated in rabbit against CEV-VP1 protein. As shown in Figure 7, strong immunoreactions were observed in the cytoplasm of the infected cells compared with the non-infected cell control group, further demonstrating the presence of enterovirus.

## Discussion

In this study, we conducted an epidemiological investigation on the newly-reported caprine/ovine enterovirus infection, and explored the infection distribution, the epidemic patterns, and the potential factors contributing to CEV/OEV infection. We found an extensive CEV/OEV infection in goat and sheep herds and the underlying contributing factors.

CEV/OEV infection is an emerging disease characterized by severe digestive and respiratory diseases that cause an important economic loss to goat industry (1–4). Currently, little information was available for its epidemiological aspects, especially on the prevalence, the distribution, the epidemic pattern, and the affecting factors. Therefore, we carried out the detection of CEV/OEV infection in goat/sheep herds using a previously established ELISA kit for detection of CEV/OEV antigens (22). We found that CEV/OEV infection was extensively detected in goat/sheep herds with a varied infection rate from different regions in China. Since the goat/sheep industry in Henan province ranks the top in China, we selected Henan to further explore the infection pattern and the factors contributing to CEV/OEV infection. Analysis of the results revealed a varied range of CEV/OEV infection in the goat/sheep herds from different regions in Henan province. The difference of infection rates in different regions might be caused by multiple factors such as geographical location, raising mode, goat or sheep breeds, different seasons etc. Comparison analysis showed that CEV/OEV infection rate was higher in diarrheal goat/sheep herds than that in non-diarrheal goat/sheep herds, indicating that CEV/OEV is likely one of the causative agents associated with diarrhea in goat and sheep. While CEV/OEV was also detected in portion of specimens collected from the clinical health goats and sheep without diarrhea, these results suggest the presence of CEV/OEV subclinical infection in goats and sheep, which is consistent with the epidemiological findings reported previously (24). Moreover, significant difference of the infection rates revealed in/among goats and sheep breeds indicated they had the different susceptibility to enterovirus infection, even within the different breeds of goats or sheep. The findings that goats or sheep raised in-house had a higher infection rate than these raised in free-range or semi-free range support the notion that raising mode did affect the enterovirus infection rate. The reason is that enclosure environment, mostly for large-scale goat/sheep farming, likely provides more opportunities for goats or sheep to contact, which promotes the spread of the virus infection. Furthermore, our investigation also showed that CEV/OEV infection had tendency to increase in recent years while the infection rate was higher in autumn and spring than those in summer and winter. This is likely due to the dramatic temperature difference between day and night in autumn and spring seasons, resulting in severe diseases with diarrhea and respiratory signs.

Our results from ELISA kit were also partially confirmed by PCR, electron microscopy, virus isolation, and immunoperoxidase monolayer assay. Parallel detection of the ELISA positive and negative specimens with PCR showed a coincidence rate of 95% between ELISA and PCR. It is surprised to note that one of the ELISA-positive specimens failed to be amplified by PCR. This may be caused by the low virus load in that positive sample or the degradation of viral RNA during the extraction process, thus leading to a false negative result. As expected, the detection coincidence rate between ELISA and electron microscopy was 100%. As for virus isolation, our results showed that enterovirus strains were only to be isolated from portion of ELISA-positive samples. This is likely due to the lack of freshness for specimens or inactivation of virus particles in the course of sample collection, shipping, and storage.

In conclusion, we investigate the epidemiological aspects of the newly-reported CEV/OEV infection, revealed the prevalence, infection distribution, epidemic pattern, and the contributing factors. The findings will provide the epidemiological theoretical basis for future prevention and control of CEV/OEV infection.

## Data availability statement

The original contributions presented in the study are included in the article/supplementary material, further inquiries can be directed to the corresponding author.

## Ethics statement

The animal study was reviewed and approved by Animal Ethics Committee of Jilin University.

## Author contributions

XW: conceptualization, writing and editing. JH, XC, and RW: epidemiology investigation and data analysis. RW and MQ: sample collection and processing. QZ and FaZ: validation. ZZ and FuZ: investigation. JH and RW: writing—original draft preparation. All authors have read and agreed to the published version of the manuscript.

## Funding

This work was supported by the National Key Research & Development Program (grant numbers: 2016YFD0500904 and 2017YFD0500104).

## Conflict of interest

The authors declare that the research was conducted in the absence of any commercial or financial relationships that could be construed as a potential conflict of interest.

## Publisher's note

All claims expressed in this article are solely those of the authors and do not necessarily represent those of their affiliated organizations, or those of the publisher, the editors and the reviewers. Any product that may be evaluated in this article, or claim that may be made by its manufacturer, is not guaranteed or endorsed by the publisher.
